# Deploying Proteins as Electrolyte Additives in Li–S Batteries: The Multifunctional Role of Fibroin in Improving Cell Performance

**DOI:** 10.1021/acsaem.2c04131

**Published:** 2023-05-31

**Authors:** Roby Soni, Damiano Spadoni, Paul R. Shearing, Dan J. L. Brett, Constantina Lekakou, Qiong Cai, James B. Robinson, Thomas S. Miller

**Affiliations:** † Department of Chemical Engineering, Electrochemical Innovation Lab, University College London, London WC1E 7JE, U.K.; ‡ The Faraday Institution, Quad One, Harwell Science and Innovation Campus, Didcot OX11 0RA, U.K.; § School of Mechanical Engineering Sciences, University of Surrey, Guildford GU2 7XH, U.K.; ∥ Department of Chemical Engineering, University of Surrey, Guildford GU2 7XH, U.K.

**Keywords:** lithium−sulfur battery, dendrites, polysulfide shuttle, molecular dynamics simulations, biomolecule

## Abstract

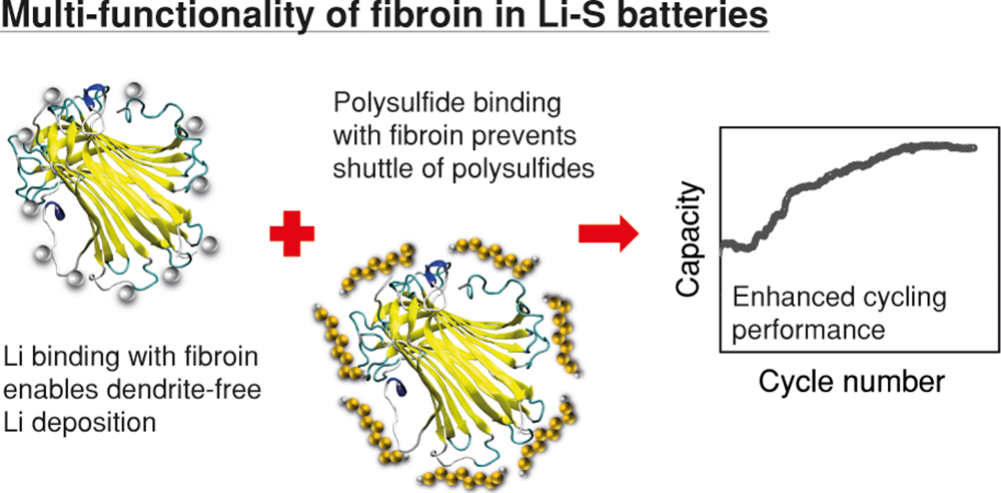

It is widely accepted that the commercial application of lithium–sulfur batteries is inhibited by their short cycle life, which is primarily caused by a combination of Li dendrite formation and active material loss due to polysulfide shuttling. Unfortunately, while numerous approaches to overcome these problems have been reported, most are unscalable and hence further hinder Li–S battery commercialization. Most approaches suggested also only tackle one of the primary mechanisms of cell degradation and failure. Here, we demonstrate that the use of a simple protein, fibroin, as an electrolyte additive can both prevent Li dendrite formation and minimize active material loss to enable high capacity and long cycle life (up to 500 cycles) in Li–S batteries, without inhibiting the rate performance of the cell. Through a combination of experiments and molecular dynamics (MD) simulations, it is demonstrated that the fibroin plays a dual role, both binding to polysulfides to hinder their transport from the cathode and passivating the Li anode to minimize dendrite nucleation and growth. Most importantly, as fibroin is inexpensive and can be simply introduced to the cell via the electrolyte, this work offers a route toward practical industrial applications of a viable Li–S battery system.

## 1 Introduction

Lithium–sulfur (Li–S) batteries have emerged as primary candidates for next-generation charge storage systems owing to their high theoretical capacity (1675 mAh/g_S_), low cost, and the relatively low environmental impact of their constituent materials, compared to alternatives.^1^ Unlike Li-ion batteries, which rely on intercalation chemistry, Li–S cells store charge through reversible redox reactions. During discharge elemental sulfur converts to Li_2_S through a series of lithium polysulfide intermediates, some of which (e.g., Li_2_S_8_, Li_2_S_6_, and Li_2_S_4_) are soluble in traditional electrolytes and some of which (e.g., Li_2_S_2_) are only partially soluble.^2^ This solubility leads to a phenomenon known as the polysulfide shuttle, where polysulfides move back and forth between the sulfur-containing cathode and the metallic Li anode due to changing concentration and potential gradients within the cell during charge/discharge. This can result in the loss of active material (i.e., sulfur inventory) via deposition of polysulfides at the anode,^3^ and hence, this polysulfide shuttle reduces the battery capacity and limits the cycle life. Together with processes promoting anode instability, such as solid electrolyte interface (SEI) growth and the development of Li dendrites,^4^ uncontrolled polysulfide shuttling has significantly hindered the commercial viability of Li–S cells.

Numerous strategies have been adopted to tackle the polysulfide shuttle effect. For example, functional carbon cathode support structures^5−7^ have been doped with heteroatoms,^8−10^ functionalized to promote M–N_*x*_ coordination, coated with metal oxides, or used alongside sulfide composites,^11−14^ all with the intention of trapping polysulfides within the cathode. Alternatively functional separators that hinder polysulfide migration from the cathode have been tested.^15,16^

Another major area of research interest concerns the development of novel electrolyte formulations that limit polysulfide dissolution.^17,18^ Additives can modify the dissolution of polysulfides and electrode/electrolyte interphase of the Li anode, therefore, can greatly impact the cycle life of Li–S cells. In particular, various methods have been used to counter anode instability, including the use of additive compounds such as LiNO_3_,^19^ fluoroethylene carbonate (FEC),^20^ LiI,^21^ P_2_S_5_,^22^ LiBr,^23^ or lithium azide.^24^ These have variously been shown to suppress polysulfide shuttling and offer Li protection via stable SEI layer formation. Unfortunately, while these electrolyte additives offer a simple and easily scalable solution for anode control, they are commonly associated with problems linked to scalability or commercial viability. For example, LiNO_3_ is consumed during cycling, limits possible discharge voltages, and would be difficult to commercialize due to gassing above 40 °C. Alternatively, while FEC has been shown to help stabilize some Li–S systems, it is mainly used in carbonate electrolytes which are not widely compatible with Li–S batteries.

Most importantly, many of the methods discussed above only act to address one of the numerous issues hindering Li–S battery lifetimes, while potentially adding significant complexity and cost to cell manufacture. As such, there is a clear scientific and commercial imperative to develop a simple, multifunctional solution to Li–S battery capacity loss and degradation.

Recently fibroin, a protein found in silks, has been tested as an electrolyte additive in Li∥Li_4_Ti_5_O_12_ cells, where it was demonstrated to hinder dendrite formation.^25^ Here, it was speculated that the fibroin acted by adsorbing onto the tips of mossy Li growths, reducing the electric field intensity and thereby slowing dendrite growth. Yet, while this theory was supported by ex- and in situ experiments, the specific interactions of the fibroin with Li were not explored by experimental or computational experiments.

The fibroin structure consists of a recurrent sequence of amino acids, including glycine, serine, and alanine, and it also has abundant amino^26^ and carboxylic and hydroxyl functional groups,^27^ to which polysulfides have an affinity; these groups have previously been shown to be useful for polysulfide trapping. In this work, fibroin is tested as a scalable, low-cost, multi-functional Li–S battery electrolyte additive as a means to both suppress the shuttling of soluble polysulfides and protect the Li anode from dendrite formation. Through both experimental methods and molecular dynamics (MD) simulations, it is shown to enhance both capacity and cycle life by mediating Li^+^ transport at the anode and acting as a polysulfide trap.

## 2 Experimental Section

### 2.1 Materials

Nanomyte BE-70 sulfur positive electrodes were procured from the NEI Corporation, USA. The electrodes, composed of 70 wt % sulfur, 10 wt % polyvinylidene fluoride binder, and 20 wt % carbon black, were used as received. The active loading of sulfur was 3.4 mg cm^–2^ (thickness 55 μm). Lithium disks (15.6 mm diameter and 0.45 mm thickness) were purchased from PI-KEM Ltd. For electrolyte preparation, 1,2-dimethoxyethane (DME), 1,3-dioxolane (DOL) solvents, lithium bis(trifluoromethanesulfonyl)imide (LiTFSI), and lithium nitrate (LiNO_3_) salts were provided by Sigma-Aldrich. Lithium sulfide and sulfur, used for polysulfide synthesis, were also procured from Sigma-Aldrich. Fibroin (Fancci, molecular weight >200,000 Da) was purchased from Simatech Inc., China.

### 2.2 Cell Fabrication

Two-electrode CR2032 coin cells were constructed by stacking a Li disk (2072% excess), separator (Celgard-2400, 25 μm), and a sulfur positive electrode, before an electrolyte containing 1 M LiTFSI and 0.8 M LiNO_3_ in a 1:1 v/v mixture of DOL/DME was added to assemble control cells. A 0.8 wt/v % fibroin electrolyte solution was prepared by adding the required amount of fibroin into the electrolyte mentioned above, before it was sonicated to disperse fibroin. Two 0.5 mm spacers and a spring (1.2 mm high and 0.3 mm thick) were used in the cell. An electrolyte to sulfur (E/S) ratio of 10 μL/mg_Sulfur_ (52 μL) was maintained in all cells. Symmetric Li∥Li cells were fabricated in a similar manner as above, but with the addition of 50 μL of electrolyte with/without fibroin.

### 2.3 Electrochemical Measurement

Charge–discharge and cyclic voltammetry measurements were performed using a BCS-805 battery cycler, whereas electrochemical impedance spectroscopy (EIS) measurements were recorded using a VSP Biologic multichannel potentiostat at room temperature. Potentiostatic EIS measurements were performed under open-circuit conditions with an applied amplitude of 5 mV. Measurements were made by scanning the frequency from 1 to 50 mHz to (swept from high to low frequency) and recording 10 points per decade for each EIS measurement. Cells were rested for 2 h postfabrication to allow electrode wetting. Before performing measurements, formation cycles were performed by first discharging the cell to 1.8 V at C/20 (*C* = 1675 mA g^–1^_Sulfur_), followed by a full charge–discharge cycle at C/20 in the voltage range of 1.8–2.6 V. The cells were kept at OCV for 45 min to allow the cell to achieve steady-state before EIS measurements were made. Lithium plating and stripping studies were done in constant current mode.

### 2.4 UV–Visible Spectroscopy Measurements

UV–Vis spectroscopy measurements were performed using a Shimadzu UV-2600 UV–VIS photospectrometer. For UV analysis, a 1 M Li_2_S_6_ solution was prepared by following a reported procedure;^28^ briefly, sulfur and Li_2_S in the appropriate molar ratio were mixed in DME by constant stirring at 60 °C to obtain a 1 M Li_2_S_6_ solution. From the above solution, 10 mM Li_2_S_6_ was prepared through dilution in DME to be used as a control sample. Similarly, to measure the UV spectrum of the fibroin-Li_2_S_6_ solution, an appropriate amount of fibroin was added to the Li_2_S_6_ solution; the final concentration of fibroin in the Li_2_S_6_ solution was maintained at 0.8 wt/v %. Pure DME was used as a blank for background correction.

### 2.5 X-Ray Photoelectron Spectroscopy Analysis

For the XPS analysis, a Thermo K-alpha spectrometer was used in constant analyzer energy mode. Photoemission was carried out using a monochromated Al K-Alpha X-ray source (1486.6 eV) with a 400 μm diameter spot size. Survey spectra were recoded using pass energy of 200 eV and the core level spectra were recorded at 50 eV pass energy. A dual beam flood gun was used to avoid sample charging. The electrodes were extracted from the cells with and without fibroin after cycling and transferred to the spectrometer using an air-free XPS transfer module (Thermo Fisher). Deconvolution and fitting of the spectra were carried out using CASA XPS software.

### 2.6 Computational Methods

MD simulations were carried out using the AMBER20 software package,^29^ with graphics processing unit (GPU) acceleration installed on the high-performance computing (HPC) clusters Augusta based at the University of Nottingham. The PACKMOL software^30^ was used to generate the initial molecular structures of the electrolyte systems in a cubic box of 294 × 294 × 294 Å^3^. Periodic boundary conditions were applied to all the three directions. Six electrolyte systems were considered for MD simulations. The initial system (System I) contained one enzyme *Bm*Fibroin (*Bombyx mori*, homotetramer, PDB ID: 3UA0)^31^ molecule (corresponding to 0.2% w/v fibroin) in a mixture of 14,666 DME molecules and 22,000 DOL molecules which correspond to a volumetric ratio of 50:50 v/v of DOL–DME. The other five electrolyte systems all contained 1 M LiTFSI (lithium bis(trifluoromethanesulfonyl)imide) added to System **I** but had different polysulfide species: no polysulfide in System **II**; 0.1 M Li_2_S_2_ in System **III**, as Li_2_S_2_ has limited solubility in DOL–DME; 0.25 M Li_2_S_4_ in System **IV**; 1 M Li_2_S_6_ in System **V**; 1 M Li_2_S_8_ in System **VI**.

AMBER ff14SB force field^32^ was used along with the generalized AMBER force field (GAFF) for nonstandard residues.^33^ Partial charges for dimethoxyethane (DME), dioxolane (DOL), bis(trifluoromethane)sulfonimide (TFSI), and polysulfide anions (S_2_^2–^, S_4_^2–^, S_6_^2–^, S_8_^2–^) were calculated employing the standard restrained electrostatic potential (RESP) protocol using Antechamber v. 17.3,^34^ and based on the vacuum electrostatic potential calculated at the HF/6-31G(d) level of theory, using Gaussian 16.^35^ All other simulation parameters necessary for the MD simulations were retrieved using the General Amber Force Field 2 (GAFF2)^33^ with relevant partial charges reported in Table SI-1. Atom charge scaling of 0.5 was applied to the ions contained in the Li_2_S_2_/LiTFSI system with fibroin to enhance the thermodynamic and dynamic properties of the molecules involved.^36,37^ The particle mesh Ewald (PME) algorithm was used to calculate long-range electrostatics, and the other nonbonded interactions have been calculated with a cut-off of 12 Å to not slow down the calculation and still include significant inter atomic connections. The system was slowly (1 ns simulation length) heated up from 0 to 300 K in an *NVT* ensemble, at constant volume. Equilibration and production steps were carried out at a constant temperature of 300 K and constant pressure of 1 atm (*NPT* ensemble). The temperature was adjusted using Langevin dynamics^24^ with a collision frequency of 1 ps^–1^, and isotropic scaling was used to maintain the pressure with a relaxation time of 2 ps. Trajectory production was performed over multiple steps of 10 nanoseconds each, until the overall simulation time scale of at least 100 ns was collected for analyzing the radial distribution function and coordination numbers.

## 3 Results and Discussion

**Figure 1:**
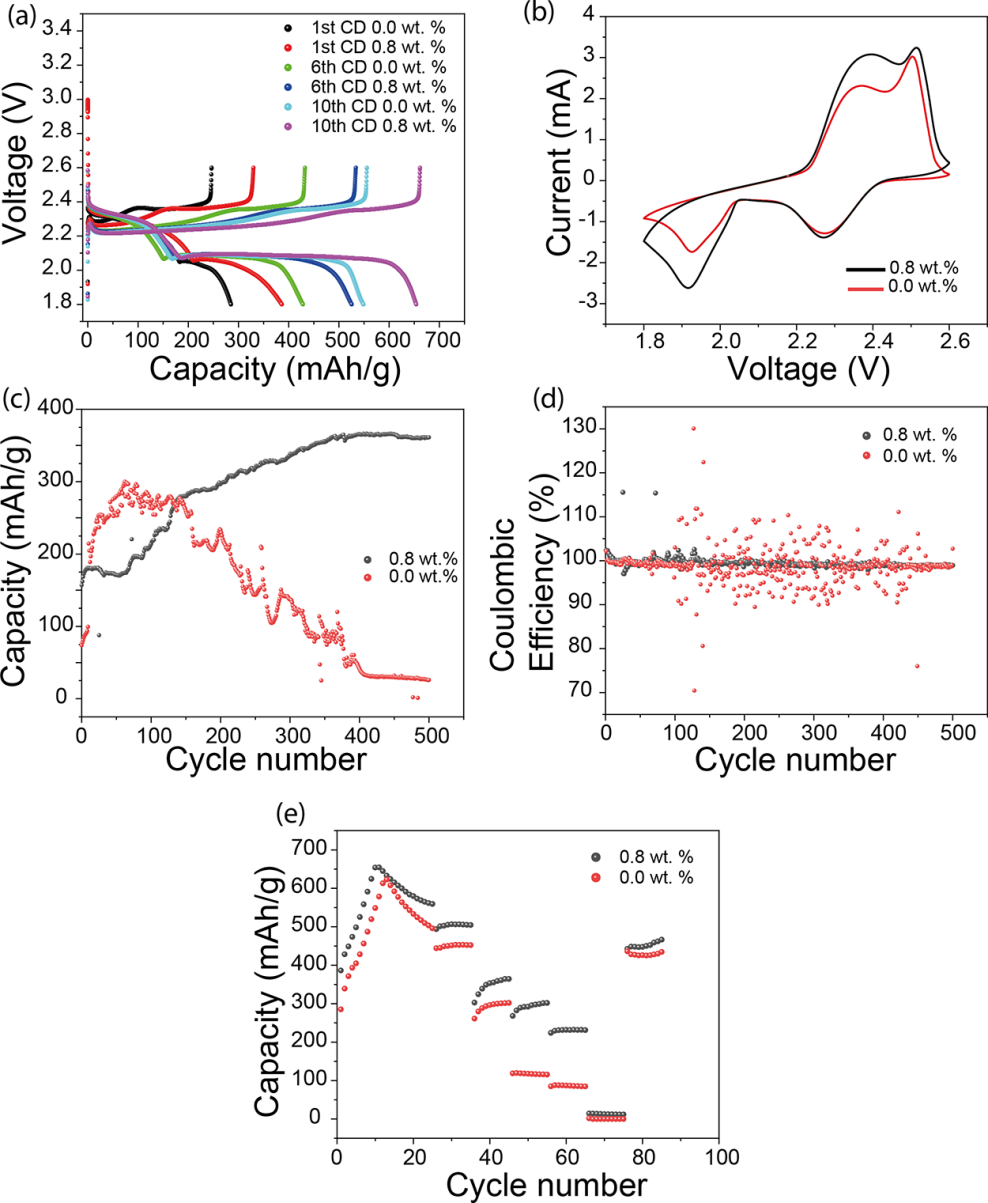
(a) Charge–discharge curves recorded at C/20 for the cells with and without fibroin; (b) CVs measured at a scan rate of 0.1 mV/s; (c) durability test performed at charge–discharge rate of C/5; (d) comparison of the Coulombic efficiency of cell with and without fibroin additives; and (e) rate capability comparison of cell with and without fibroin.

Electrochemical characteristics of Li–S cells containing electrolytes with and without 0.8 wt % fibroin are shown in Figure 1. This concentration was chosen as it was close to the dissolution limit of fibroin in the electrolyte without leading to turbid solutions. Charge–discharge profiles measured at a rate of C/20 (Figure 1a) show a consistently higher capacity for a cell containing fibroin compared to a control cell; after the first discharge, the fibroin cell showed a capacity of 386 mAh/g (control cell 285 mAh/g), and after the 6th and 10th cycles, the fibroin cell showed capacities of 525 and 654 mAh/g (428 and 548 mAh/g for the control cell), respectively. This higher capacity points toward significantly increased sulfur utilization. Furthermore, it can be observed that the second plateau in the charge–discharge, representing the formation of Li_2_S_2_ from the polysulfides, is significantly longer in the cell-containing fibroin, which indicates the presence of high concentration of polysulfides at the positive electrode interface for conversion to Li_2_S. Cyclic voltammograms (CVs) recorded at a scan rate of 0.1 mV/s (Figure 1b) confirm the increased conversion efficiency of polysulfides to Li_2_S_2_ in the fibroin containing electrolyte via the increased current density in the 1.8–2.0 V region. As the sulfur loading is the same in the two cells, this high polysulfide conversion current in the electrolyte with the fibroin additive suggests a strong binding of polysulfides with fibroin at the cathode interface, indicating that it prevents polysulfide shuttling and therefore the loss of polysulfides to the electrolyte/anode.

Durability assessment of the fibroin-containing cell and a control, carried out at a discharge rate of C/5, is shown in Figure 1c, where it can be seen that the addition of fibroin improved the long-term cycling performance. The cell containing fibroin showed little capacity loss over 500 charge–discharge cycles, and in fact between cycle 1 and 377, the cell capacity actually increased significantly from 175 to 364 mAh/g. It should be noted that the commercial nanomyte cathodes offer significant benefits in terms of reproducibility, but at a lower capacity than laboratory-made electrodes. These electrodes also routinely present a characteristic increase in accessible capacity through the first 75 cycles at higher C rates.^38^ In comparison the control cell reached a maximum capacity of 300 mAh/g after just 75 cycles and remained relatively stable up to 180 cycles, before exhibiting a drastic decrease in capacity to less than 50 mAh/g after 400 cycles. Here, in the fibroin containing cells, the presence of protein at the active material interfaces likely contributed to a slower initial utilization of sulfur, which may explain the gradual increase in capacity observed in the early cycles, but ultimately led to a more stable cathode structure (as discussed below). As a primary cause of capacity loss in Li–S cells is loss of active material due to the polysulfide shuttle;^39,40^ these data further suggest that the fibroin acts to hinder polysulfide mobility.

Equivalent durability data measured at C/5 for 0.3 and 0.5 wt % fibroin (Figure SI-1) suggest that lower concentrations of fibroin may help stabilize the cells, but to a lesser degree than the 0.8 wt % case.

Higher and more consistent Coulombic efficiencies of the fibroin-containing cells (∼99%) throughout their cycle life (Figure 1d) demonstrate that these cells are less prone to parasitic losses than fibroin free equivalents. Interestingly the fibroin-containing cell also showed better rate performance, when compared to the control cell (Figure 1e), exhibiting a capacity of 301 mAh/g at C/3 and 232 mAh/g at C/2, compared to 115 and 85 mAh/g at the same rates for the fibroin free cell; suppression of polysulfide dissolution and migration are most likely responsible for high-rate capability in fibroin cells.^41,42^ Importantly, similar trends were observed in repeat experiments (Figure SI-2).

To gain an initial insight into the improved cycle life of fibroin-containing cells, we conducted post-mortem scanning electron microscopy (SEM) analysis of cathodes recovered from cycled cells. SEM images of a cathode extracted from a cell containing 0.0 wt % fibroin (Figure SI-3a,b) revealed a thick and insulating film deposited on the cathode surface, which can be attributed to the formation of electrically isolated Li_2_S at the electrode interface due to polysulfide shuttling. In contrast, SEM analysis of an electrode extracted from cells containing 0.8 wt % fibroin (Figure SI-3c,d) showed a thinner and less dense interfacial film. Moreover, the active material particles did not exhibit significant agglomeration or Li_2_S inhibition on their surfaces, suggesting that the incorporation of fibroin hinders the formation of insulating films on the cathode surface.

**Figure 2:**
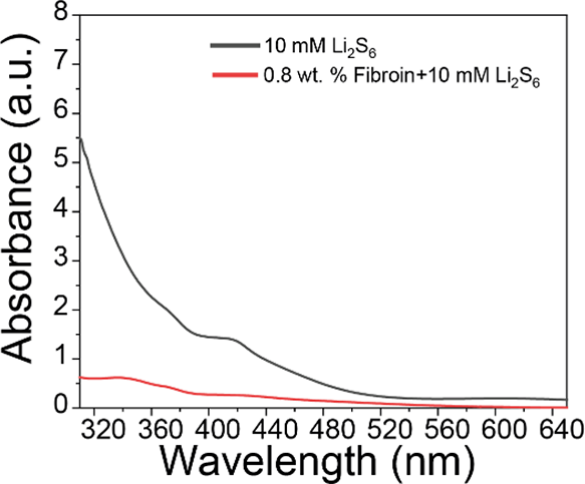
UV–visible spectra of 10 mM Li_2_S_6_ solution in DME in the absence and presence of fibroin.

To further explore the role of fibroin in improving the durability, cyclability, and capacity of Li–S cells, experimental characterization was combined with MD simulations. UV spectra of 10 mM Li_2_S_6_ in 1,2-dimethoxyethane, and an equivalent solution containing 0.8 wt % fibroin, are shown in Figure 2. The pure Li_2_S_6_ solution showed a very high absorbance from 320 to ∼520 nm, which decreased drastically with the addition of fibroin, from 5.5 to ∼0.5 (a.u.) at 320 nm, which indicates strong anchoring of the polysulfide onto the fibroin molecules. Polysulfides have been found to strongly bind with nitrogen-doped carbon^43^ and small organic molecules^44^ containing nitrogen and oxygen; as fibroin is also abundant in oxygen and nitrogen, it should be able to strongly bind with polysulfides (also see MD results below). This explains the high current density in the CVs (Figure 1b), as the mass transport of polysulfides bound to fibroin would be hindered, and therefore, their escape from the positive electrode would be more difficult; hence, they would be available for conversion back to S upon charging. In the control cell, the polysulfides could freely diffuse into the electrolyte and get lost at the anode.

**Figure 3:**
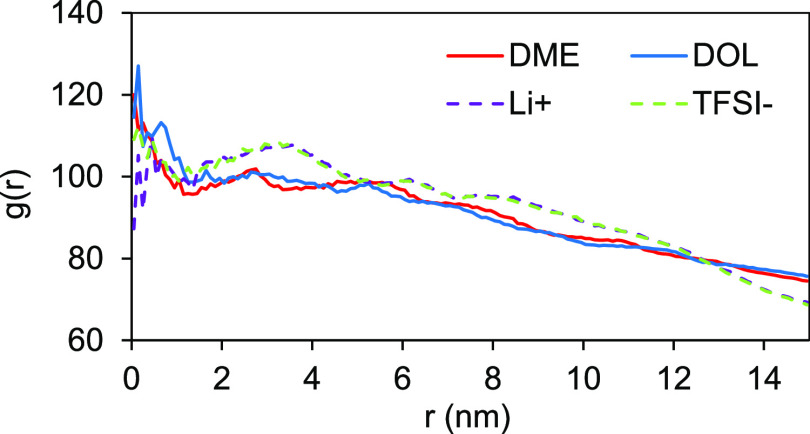
Center-of-mass RDF between the center of fibroin structure, solvent molecules DME and DOL, and ions Li^+^ and TFSI^–^.

MD simulations were performed to gain an insight into the mechanisms driving polysulfide binding in the presence of fibroin in Li–S cells, but first, it was necessary to determine how fibroin interacts with traditional Li–S battery electrolyte components. Simulations of System I (0.2% w/v fibroin in DOL–DME 50:50 v/v, Figure SI-5d) show that the fibroin structure becomes impregnated by both DOL and DME upon exposure, highlighting its affinity with Li–S electrolytes, forming a dual solvation structure comprising an inner DOL shell of ∼4 nm diameter and an outer DME shell of ∼8 nm diameter. Importantly, when Li^+^ is added into the system (System II: 0.2% w/v fibroin, 1 M LiTFSI in DOL–DME 50:50 v/v, Figures 3 and SI-7), it can be seen that that Li^+^ ions also impregnate the fibroin structure, with a radial distribution function, *g*(*r*), maximum within a diameter of 1 nm and adsorb at the outer surface of the protein structure with a maximum *g*(*r*) within a diameter of 3 nm. This high affinity of Li^+^ ions to the protein structure is attributed to the creation of secondary lithium bonding, as in −CO···Li^+^, and interactions with the electronegative part of the fibroin polar amino acids serine and tyrosine. Figure 3 shows that TFSI^–^ ions also impregnate the fibroin structure and adsorb at the outer surface, which is attributed to the interactions with the electropositive parts of these polar amino acids. These interactions stabilize the protein structure and reduce the root mean square deviation (RMSD) and route mean square fluctuation (RMSF), when comparing the average structure in System II (Figure SI-7) to that in System I (without LiTFSI). This again confirms that the fibroin will influence the electrolyte structure and hence direct its properties. Figure 3 and Tables SI-2 and SI-3 also show that the protein in the presence of 1 M LiTFSI absorbs more DME and DOL, possibly in the form of solvated TFSI^–^ and Li^+^ ions.

The impregnation and coordination number (*N*_I+C_, in Table SI-2) and the coordination number of the solvation shell (*N*_C_, in Table SI-3) of the different system species with respect to the quaternary structure of the fibroin protein further confirm that for the six electrolyte systems simulated, the silk fibroin protein is highly solvated in the DOL/DME solution, with a solvated sphere diameter of about 15 nm (Figure SI-5d). The protein absorbs a large number of Li^+^ ions (*N*_I+C_ = 617 for System II), which possibly control the concentration gradients at the anode interphase and hence Li-plating behavior, by distributing Li^+^ ions between the anode surface and the nearby fibroin molecules, which would slow down the rate of Li^+^ ion plating on the anode and reduce dendrite formation.

**Figure 4:**
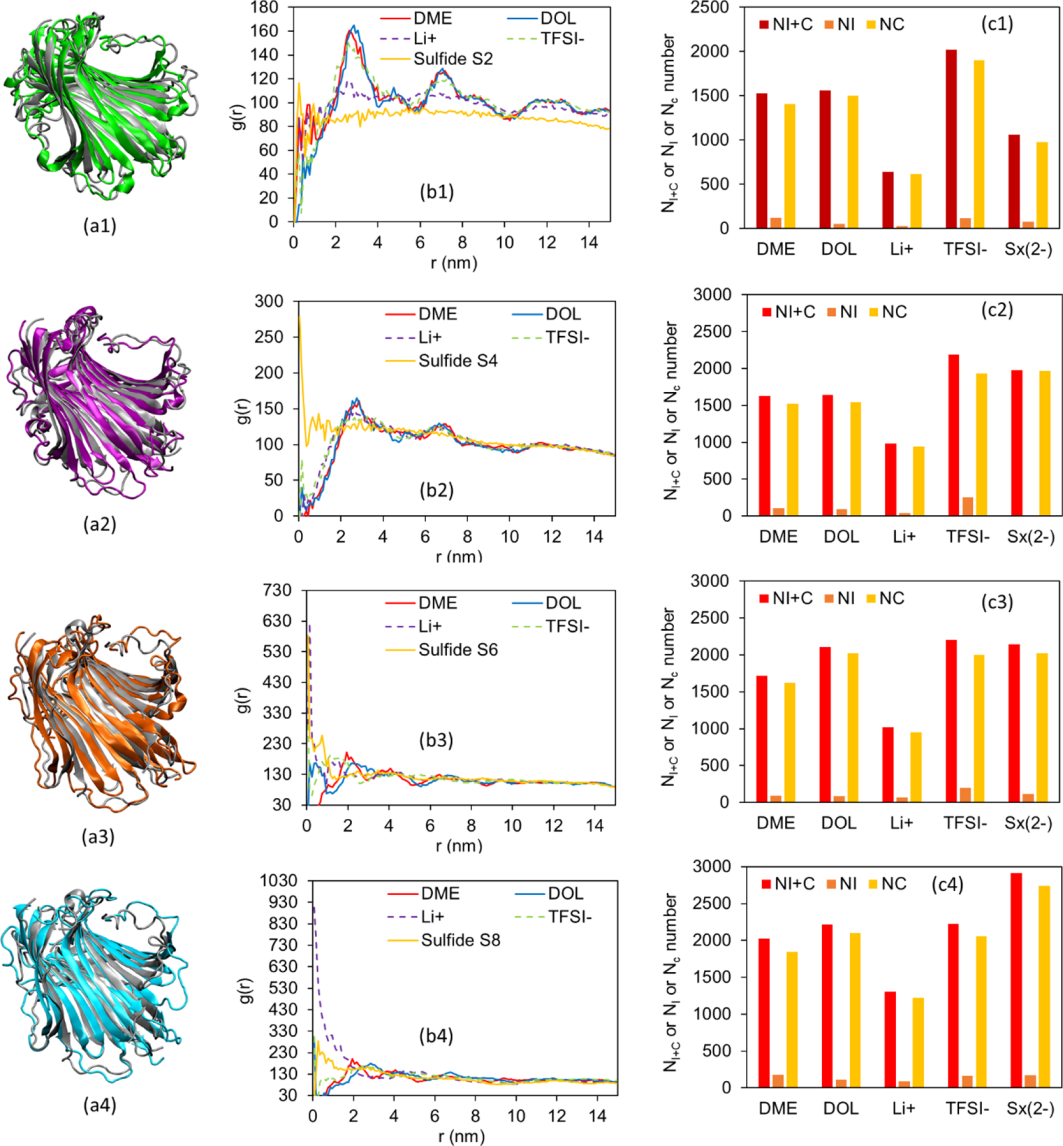
MD simulation results for (0.2% w/v fibroin, 1 M LiTFSI in DOL–DME 50:50 v/v and Li_2_S_*x*_ as follows: (1) System III: 0.1 M Li_2_S_2_; (2) System IV: 0.25 M Li_2_S_4_; (3) System V: 1 M Li_2_S_6_; (4) System VI: 1 M Li_2_S_8_. Types of figures: (a1–a4) fibroin structure in the current system with Li_2_S_*x*_ (colored) versus that in System I (gray); (b1–b4) center-of-mass RDF between the center of the fibroin structure, solvent molecules DME and DOL and ions Li^+^, TFSI^–^, and polysulfide S_*x*_^2^ in the corresponding system; (c1–c4) impregnation and coordination number, *N*_I+C_, impregnation number *N*_I_ and coordination number *N*_C_ of solvent molecules DME and DOL and ions Li^+^, TFSI^–^, and polysulfide S_*x*_^2–^ around the fibroin structure in systems 1–4, respectively.

The addition of Li_2_S_2_ to the electrolyte (System III: 0.2% w/v fibroin, 1 M LiTFSI, 0.1 M Li_2_S_2_ in DOL–DME 50:50 v/v) was found to promote Li_2_S_2_ agglomerates, as illustrated in Figure SI-8, demonstrating the low solubility of Li_2_S_2_ in DOL–DME. Yet, the Li_2_S_2_ can still be seen to induce structural differences in fibroin (System III, Figure 4a1). The RDF in Figure 4b1 describes a different scenario compared to the previous MD simulations analyzed. Peaks of *g*(*r*) near the center, i.e., near *r* = 0, show that among all the species involved, polysulfide ions, S_2_^2–^, seem to be the most likely to be found at closest distances to the fibroin center of mass, highlighting their affinity to the fibroin as supporting the experimental data, while all the other species show a first solvation shell at a radius, *r*, between 3 and 7 nm. The strong affinity of fibroin to Li^+^ cations and S_2_^2–^ anions is supported by the high coordination number (*N*_C_) for S_2_^2–^ and Li^+^ ions (Table SI-3 and Figure 4c1) around the fibroin structure (despite the low overall concentration of Li_2_S_2_ at 0.1 M compared with 1 M LiTFSI) and the *N*_I+C_ number which also shows impregnation of these ions in the fibroin structure. The *N*_C_ and *N*_I+C_ numbers of the solvents DOL and DME are even greater for System III, compared to Systems I and II, as additional solvent molecules are present to solvate the polysulfide ions.

MD simulations for System IV (0.2% w/v fibroin, 1 M LiTFSI, 0.25 M Li_2_S_4_ in DOL–DME 50:50 v/v), System V (0.2% w/v fibroin, 1 M LiTFSI, 1 M Li_2_S_6_ in DOL–DME 50:50 v/v) and System VI (0.2% w/v fibroin, 1 M LiTFSI, 1 M Li_2_S_8_ in DOL–DME 50:50 v/v), which are presented in Figures 4 and SI-10–SI-12, follow similar trends as System III (Li_2_S_2_) but without polysulfide agglomeration, given the better solubility of the higher-order polysulfides in DOL–DME. Interestingly, these data allow general trends to be identified. Figure 4a1–4 illustrate that higher-order polysulfides (up to Li_2_S_6_) tend to induce more swelling of the fibroin structures, which is confirmed by the maxima in RMSD plots in Figures SI-10–SI-12. This is attributed to the larger absorption of species in the fibroin structure, confirmed by the total impregnation number *N*_I_ of all species calculated from Tables SI-2 and SI-3, which increases from System I to System VI (*N*_I_ = 93, 325, 395, 499, 563, 719, respectively). A high *g*(*r*) peak is seen in the RDF of S_4_^2–^ (System IV, Figure 4b2), especially S_6_^2–^ (System V, Figure 4b3), and S_8_^2–^ (System VI, Figure 4b4) at the center of the fibroin structure, denoting significant deep impregnation for these three polysulfides, meaning the soluble higher-order polysulfide molecules are trapped within the fibroin structure to prevent them from going to anode. Overall, S_4_^2–^, S_6_^2–^, and S_8_^2–^ exhibit the highest coordination numbers and high impregnation numbers (Tables SI-2 and SI-3 and Figure 1c2–c4) against fibroin (the trend showing that *N*_I+C_ and *N*_C_ increase with the length of sulfide) which demonstrate the great ability of fibroin to trap polysulfides, especially the most troublesome soluble polysulfides. Importantly, this strong tendency towards polysulfide binding is expected to suppress their shuttling by holding the generated species within, or close to, the cathode structure: mass transport of the bulky fibroin/polysulfide complexes away from the cathode is expected to be slow, given their large diameter of ∼20 nm in their swollen state (see Systems III–VI in Figure 4b1–4).

Interestingly, for the electrolyte systems (System III, IV, V, VI) with polysulfides (Li_2_S_2_, Li_2_S_4_, Li_2_S_6_, and Li_2_S_8_), the impregnation and coordination numbers of Li^+^ are smaller than those of TFSI^–^ in Table SI-2, giving a *N*_I+C_ (Li^+^)/*N*_I+C_ (TFSI^–^) ratio of <1.0. This is different from System II (with no polysulfides) where the *N*_I+C_ (Li^+^)/*N*_I+C_ (TFSI^–^) ratio is 1.0. This indicates that in practical Li–S batteries with polysulfides in the electrolyte solutions, fibroin will both act to trap polysulfide species, and in doing so, regulate Li^+^ transport by releasing additional Li^+^ ions into the electrolyte upon polysulfide uptake, improving the mass transport of Li^+^ ions. A low *N*_I+C_ (Li^+^)/*N*_I+C_ (TFSI^–^) ratio would generate a high transference number for Li^+^ ions during the production of sulfides, which favors their transport to the cathode via the drift current^46^ during discharge, promoting the redox reaction cascade and increasing the cell capacity, as evidenced in Figure 1.

While the MD simulations indicate that fibroin can modulate the solvent structure of Li ions, it is also important to understand how it interacts with the solid Li anode. First operando optical microscope analysis was performed in symmetric Li∥Li cells containing 50 mM Li_2_S_*x*_ in the electrolyte, with and without fibroin. The cells were cycled at a current density of 3 mA/cm^2^, maintaining a charge capacity of 3 mAh/cm^2^ for 10 plating/stripping cycles. The results, which are presented in Figure SI-13, reveal that the incorporation of fibroin into the electrolyte solution effectively hinders dendrite formation on lithium. After 10 plating/stripping cycles, the cell without fibroin displayed severe dendrite growth (Figure SI-13b), ultimately leading to a short circuit, while the cell containing 0.8 wt % fibroin (Figure SI-13d) remained significantly more free of dendrite growth, and short circuits were avoided.

**Figure 5:**
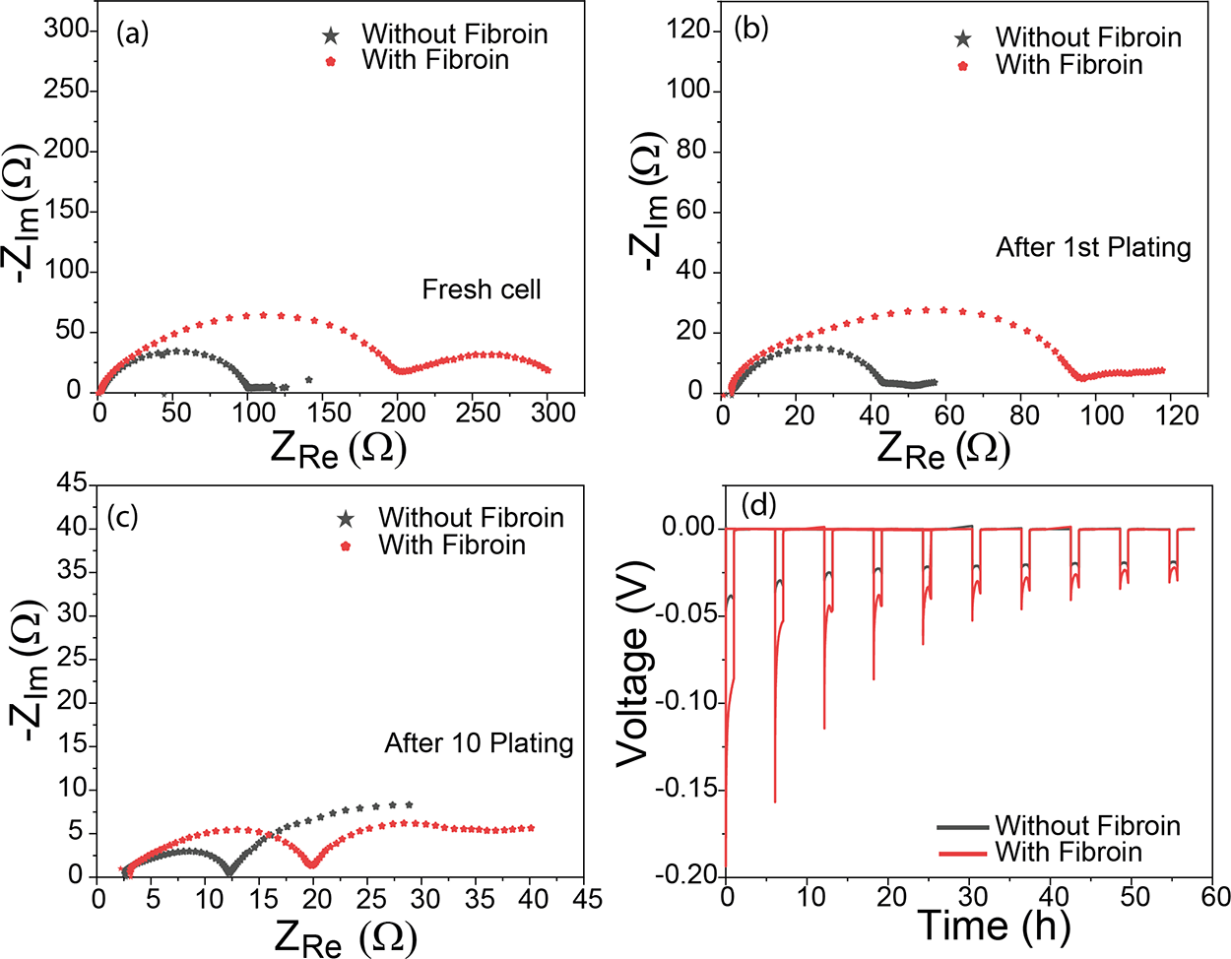
(a–c) Nyquist plot for symmetric Li cells with and without fibroin in the pristine cell, after first plating, and 10 plating cycles, respectively; (d) overpotential changes during Li plating for the initial 10 cycles.

Next, electrochemical impedance spectroscopy (EIS) measurements of symmetric Li∥Li cells were performed. First, EIS of fresh cells (i.e. in the pristine state before any electrochemical cycling) containing electrolytes with and without fibroin are shown in Figure 5a, where the equivalent series resistance (ESR) was measured to be 2.13 Ω for the fibroin cell, marginally higher than the cell without fibroin (ESR 1.93 Ω). This indicates that the added fibroin had little adverse effect on key electrolyte properties, most importantly viscosity (ESR increases significantly with increases in electrolyte viscosity). The cell containing fibroin showed a comparatively large semi-circle in the high-frequency region and high impedance in the diffusion region, compared to the cell without fibroin, indicating the presence of an additional interfacial impedance within the cell. This may suggest the fibroin’s presence at the electrode–electrolyte boundary. After the first Li plating (Figure 5b), the impedance of both cells reduced to almost half, attributed to the formation of high surface area Li film after plating; however, the cell containing fibroin still showed higher impedance than the cell without fibroin, suggesting its continued presence as a pacifying layer at the Li anode interface, a trend which continues even after 10 Li plating cycles (Figure 5c). It should however be noted that after 10 cycles, the size of a high-frequency semi-circle fell to 20 Ω in the fibroin cell and 12 Ω in the control cell, indicating continuous evolution of the electrode/electrolyte interphase. Voltage profiles of the Li plating over the initial 10 cycles can be seen in Figure 5d and show that the overpotential during the first plating was high for both cells, although the addition of fibroin almost doubled the magnitude of this initial overpotential. Over time, the overpotentials showed a gradual decrease, eventually becoming approximately equivalent and demonstrating that the initial overpotential increase is an acceptable trade-off for long-term cell stability. This increase in overpotential and impedance suggests that alongside modulating Li ion behavior, the fibroin acts to form an interfacial layer at the Li metal which pacifies kinetically favorable Li deposition sites (e.g., dendrites), which is consistent with the behavior noted for lithium ion cells.^25^

**Figure 6:**
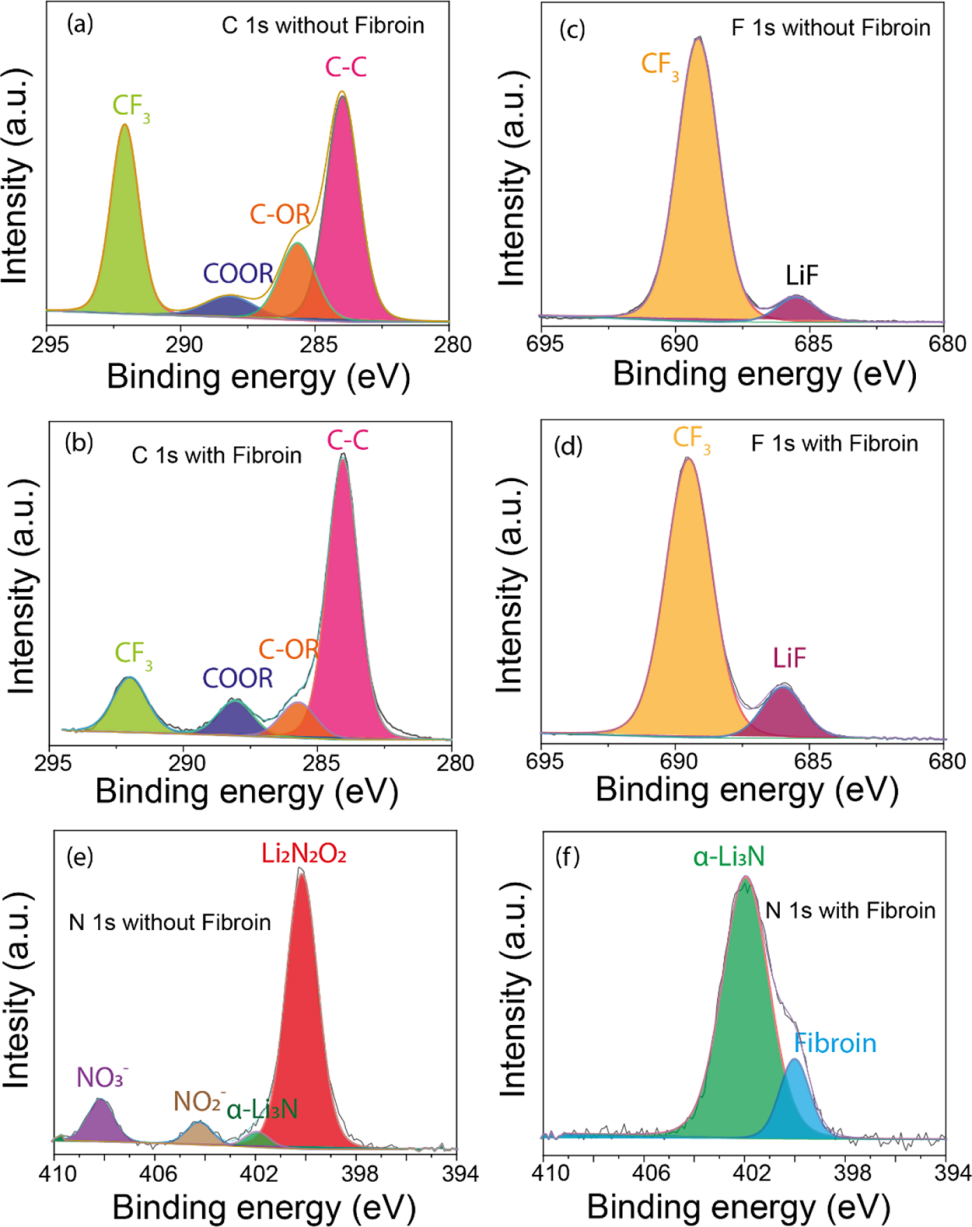
XPS analysis. (a and b) C 1s spectra of the Li anode extracted from the Li–S cells without and with fibroin, respectively; (c and d) F 1s spectra measured for the Li anode for the Li–S cells without and with fibroin, respectively; (e and f) N 1s spectra measured for the Li anode for the Li–S cells without and with fibroin, respectively.

XPS measurements of Li anodes extracted from cycled cells with and without fibroin showed marked differences in their SEI composition. Comparison of the C 1s spectra (Figure 6a,b) from the fibroin-containing and control cell showed a significant difference in the ratio between C–C groups (∼284 eV) and CF_3_ arising from the degradation of LiTFSI (∼292 eV). A lowered concentration of CF_3_ was therefore indicative of less solvent breakdown at the anode, indicating that it has been otherwise stabilized. F 1s spectra (Figure 6c,d) corroborated this low CF_3_ content, showing a lowering of the ratio compared to LiF (686 eV), which is known to be associated with high stability and high ionic conductivity SEI structures.^45^ N 1s spectra from a cell without fibroin (Figure 4d) showed a significant presence of Li_2_N_2_O_2_ (400 eV), α-Li_3_N (402 eV), NO_2_ (404 eV), and NO_3_ (408 eV) groups^47^ which are known degradation products of LiNO_3_. Alternatively, an N 1s spectrum from a fibroin containing cell showed a large area peak at 402 eV, attributed to α-Li_3_N. Another peak at 400 eV could be primarily assigned to the nitrogen-containing groups within fibroin,^25^ although the total absence of Li_2_N_2_O_2_ cannot be guaranteed. Importantly, these data show that the SEI formed on the anode in the fibroin containing cell nitrogen species contained significantly more Li_3_N (84% of the N contribution compared to 3.6%), which is known to regulate Li nucleation, suppress dendrite formation, and enhance Coulombic efficiency owing to its high Young’s modulus.^46^

## 4 Conclusions

**Figure 7:**
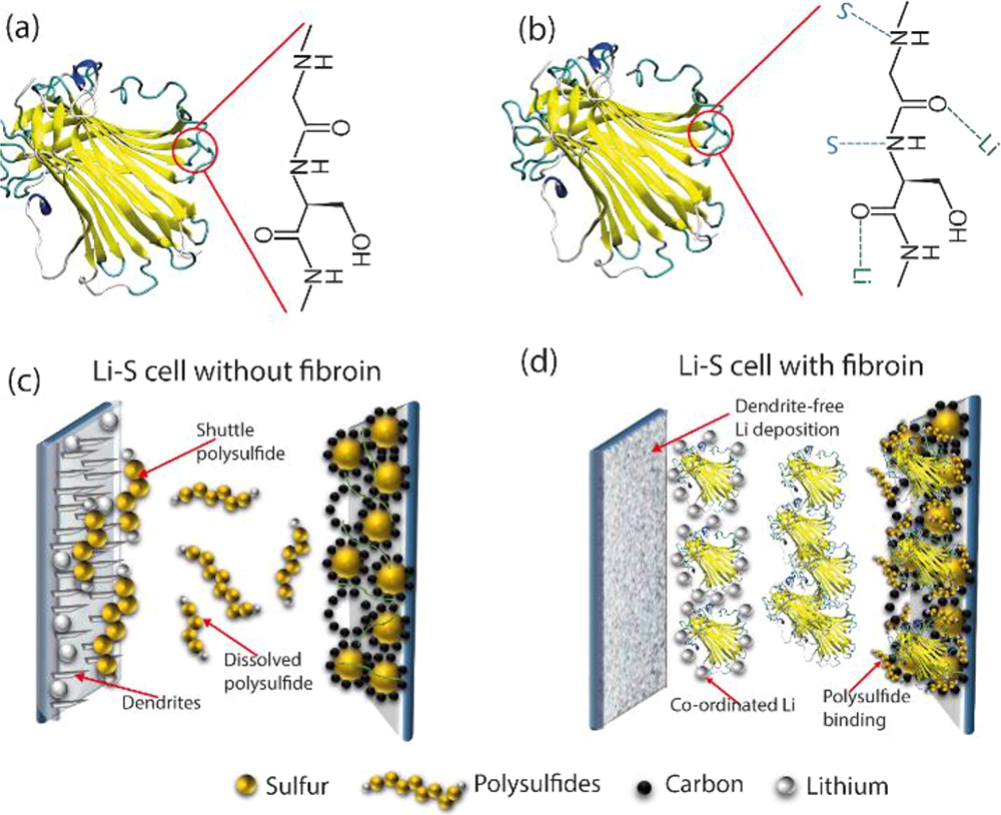
(a) Structure of the fibroin and the functional groups present in its amino acid backbone; (b) schematic representation of the interaction of fibroin functional groups with Li ions and sulfur within polysulfides when present in the Li–S electrolyte; (c) schematic representation of dendrite formation and polysulfide shuttle processes in typical Li–S cells; and (d) schematic showing the impact of fibroin as an electrolyte additive, strongly binding polysulfides at the positive electrode, controlling the polysulfide shuttle, and significant coordination of Li-ions with fibroin at the negative electrode, which mediates Li ion diffusion, resulting in dendrite-free Li deposition.

In summary, this report illustrates the application of fibroin, a common and widely available protein, as a multi-functional electrolyte additive in Li–S batteries (Figure 7). It is demonstrated that this biomolecule has the ability to trap lithium polysulfides within the cathode, minimizing sulfur losses and maximizing utilization, and that its reported ability to hinder the nucleation and growth of dendrites extends to Li–S battery anodes. Cells containing fibroin showed high capacity, good rate capability, and long cycle lifetimes compared to equivalent control cells.

Through a combination of experimental measurements and MD simulations, we have developed a detailed understanding of the mechanisms underlying interactions between fibroin, polysulfides and Li. Combined UV–Vis spectroscopy and simulations have shown that any polysulfide generated will be strongly held within the fibroin, becoming deeply impregnated within the protein structure, preventing mass transport from the cathode to the anode. Importantly the most soluble, and therefore most important to control, polysulfides (Li_2_S_8_, Li_2_S_6_, and Li_2_S_4_) exhibit a particular affinity to bind with fibroin. At the Li anode, a combination of EIS and XPS highlighted that fibroin can also bind at the anode, as an artificial solid electrolyte interphase layer, mitigating the nucleation and growth of dendrites. Together, this work has shown that a simple and scalable electrolyte additive can stabilize Li–S batteries, offering a route toward commercial viability for this technology.

## Supporting Information Available

Protocols for MD simulations; cycling data with different fibroin contents; electrochemical data of fibroin and baseline cells; scanning electron microscopy images; MD simulation results; optical microscope images of Li∥Li cells; impregnation and coordination number data (PDF)
