# Supplementary material for: Deploying Proteins as Electrolyte Additives in Li–S Batteries: The Multifunctional Role of Fibroin in Improving Cell Performance
Source: ACS Appl Energy Mater. 2023 May 31;6(11):5671–80. doi: 10.1021/acsaem.2c04131 (PMC10266332; doi:10.1021/acsaem.2c04131)
Supplement: Supplementary file 1 — ae2c04131_si_001.pdf [file ae2c04131_si_001.pdf]

Supporting Information

Deploying Proteins as Electrolyte Additives in Li-S Batteries: The Multifunctional Role of Fibroin in Improving Cell Performance

Roby Soni[1, 2], Damiano Spadoni[2,3,4], Paul R. Shearing[1,2], Dan J.L. Brett[1,2], Constantina Lekakou[2,3], Qiong Cai[2,4], James B. Robinson[1,2*] Thomas S. Miller[1,2*]

[1]Department of Chemical Engineering, Electrochemical Innovation Lab, University College London, London WC1E 7JE, UK

[2]The Faraday Institution, Quad One, Harwell Science and Innovation Campus, Didcot OX11 0RA, UK

[3]School of Mechanical Engineering Sciences, University of Surrey, Guildford GU2 7XH, UK

[4]Department of Chemical Engineering, University of Surrey, Guildford GU2 7XH, UK

Emails: j.b.robinson@ucl.ac.uk, t.miller@ucl.ac.uk

Protocol for the MD simulations

Table SI-1. Atom types and partial charges employed for the different species in the electrolyte.

Atom	Atom type	Partial charge
TFSI		
F1	f 1	-0.103080
C1	c3	0.173663
S1	s6	1.029770
N1	ne	-0.682273
S2	sy	1.029770
C2	c3	0.173663
F2	f	-0.103080
F3	f	-0.103080
O1	o	-0.526529
O2	o	-0.526529
O3	o	-0.526529
O4	o	-0.526529
F4	f	-0.103080
F5	f	-0.103080
F6	f	-0.103080
S_2^{2-}		
S1	s	-0.500000
S2	s	-0.500000
S_4^{2-}		
S1	s	-0.772084
S2	ss	-0.227916
S3	s	-0.772084
S4	ss	-0.227916
S_6^{2-}		
S1	s	-0.637134

S2	ss	-0.169573
S3	ss	-0.193293
S4	ss	-0.193293
S5	ss	-0.169573
S6	s	-0.637134
S_8^{2-}		
S1	s	-0.839771
S2	ss	0.237729
S3	ss	-0.049538
S4	ss	-0.348420
S5	ss	-0.348420
S6	ss	-0.049538
S7	ss	0.237729
S8	s	-0.839771

Experimental results

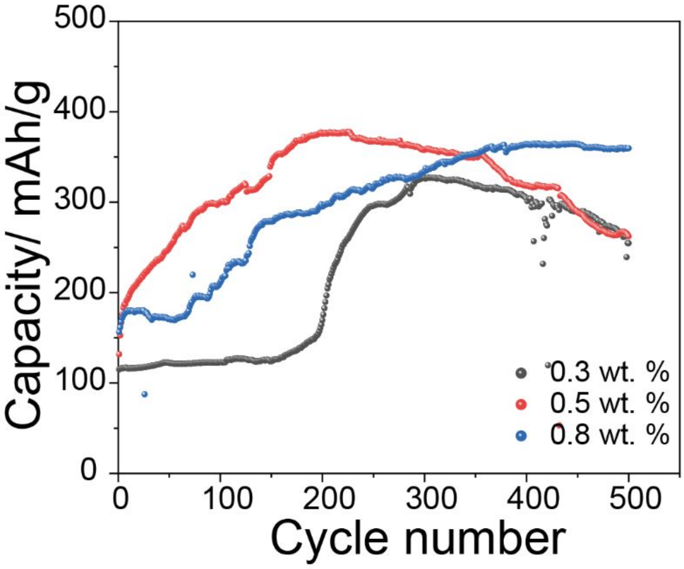

Figure SI-1. Cycling data of Li-S cell with different concentration of fibroin in the electrolyte carried out at C/5 rate.

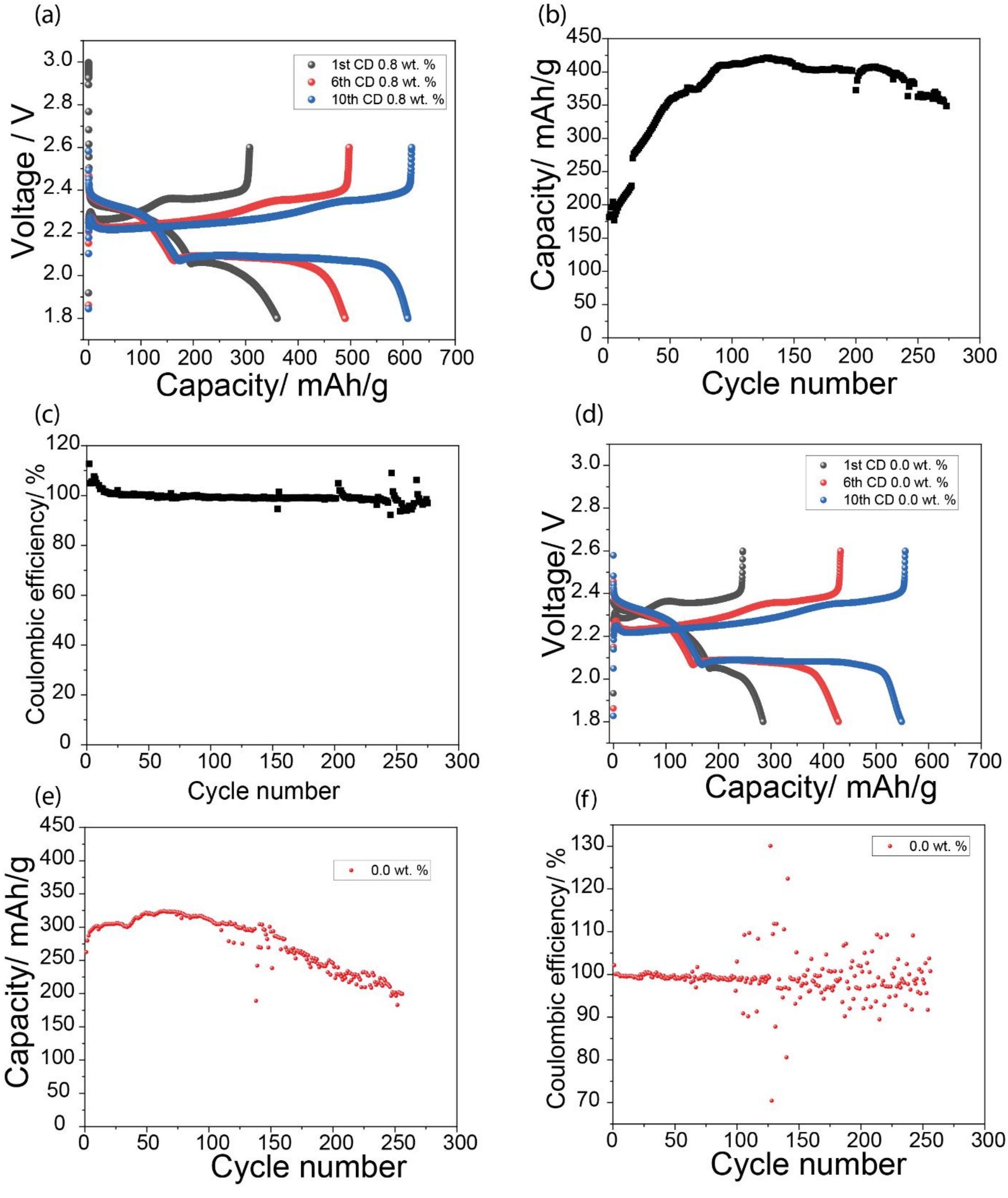

Figure SI-2. (a), (b) and (c) are the charge-discharge (C/20), stability (at C/5) and Coulombic efficiency data of Li-S cell containing fibroin. Whereas **(**d), (e) and (f) are the charge-discharge (C/20), stability (at C/5) and Coulombic efficiency data of Li-S cell without fibroin.

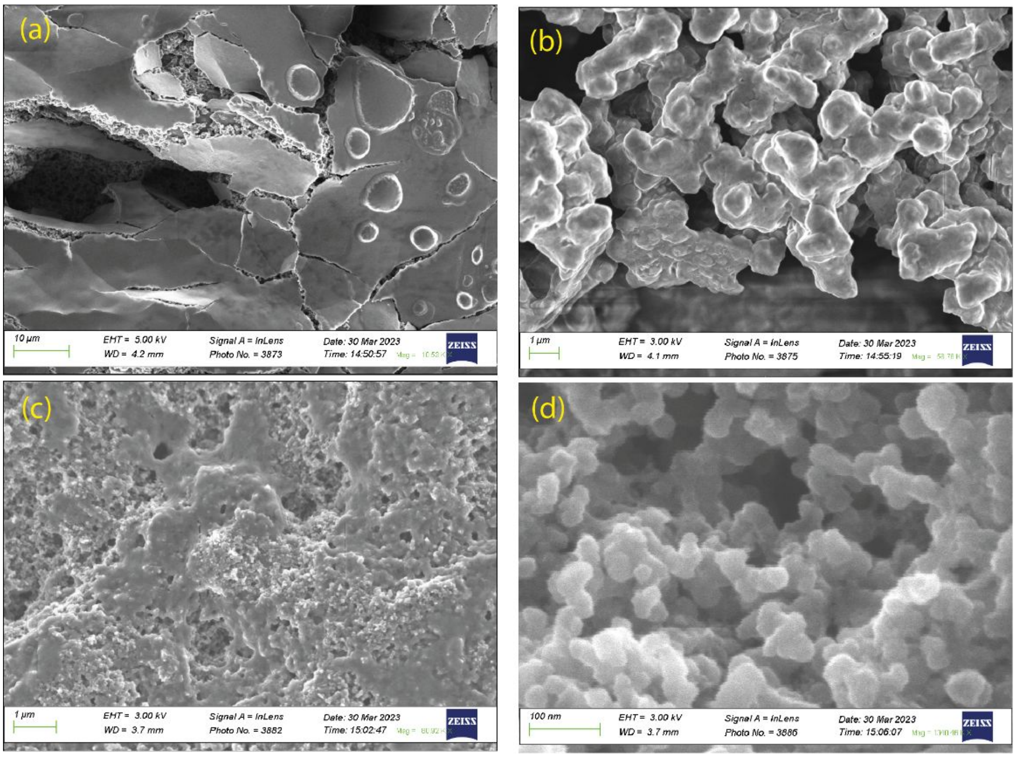

Figure SI-3. SEM analysis. (a), (b) are the SEM images of cathodes extracted from cells without fibroin after durability test; (c) and (d) SEM images of cathode recovered from cell with 0.8 wt. % fibroin after durability test.

Results of MD simulations

SYSTEM I: 0.2 % w/v fibroin in DOL:DME 50:50 v/v

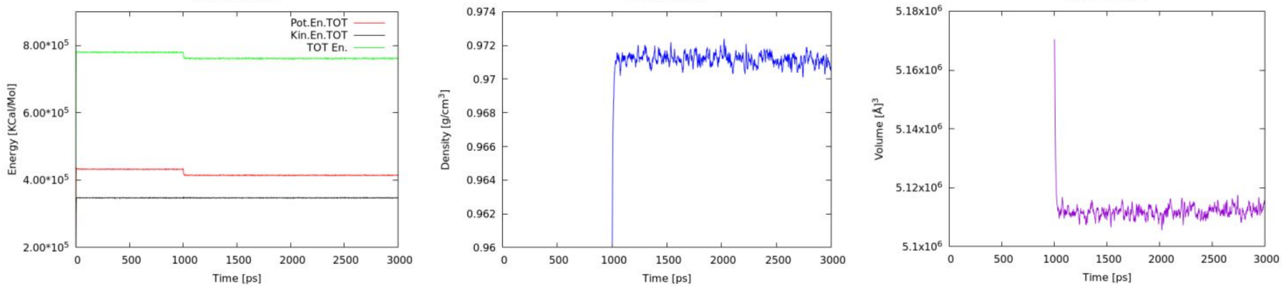

(a) (b) (c)

Figure SI-4. Plots of (a) system energies (b) system density and (c) system volume monitored during the first 1000 ps (1 ns) where the system was heated up starting from 0° to 27°C (300 K) and the equilibration step where the system was left equilibrating before starting the actual trajectory production (2000 ps).

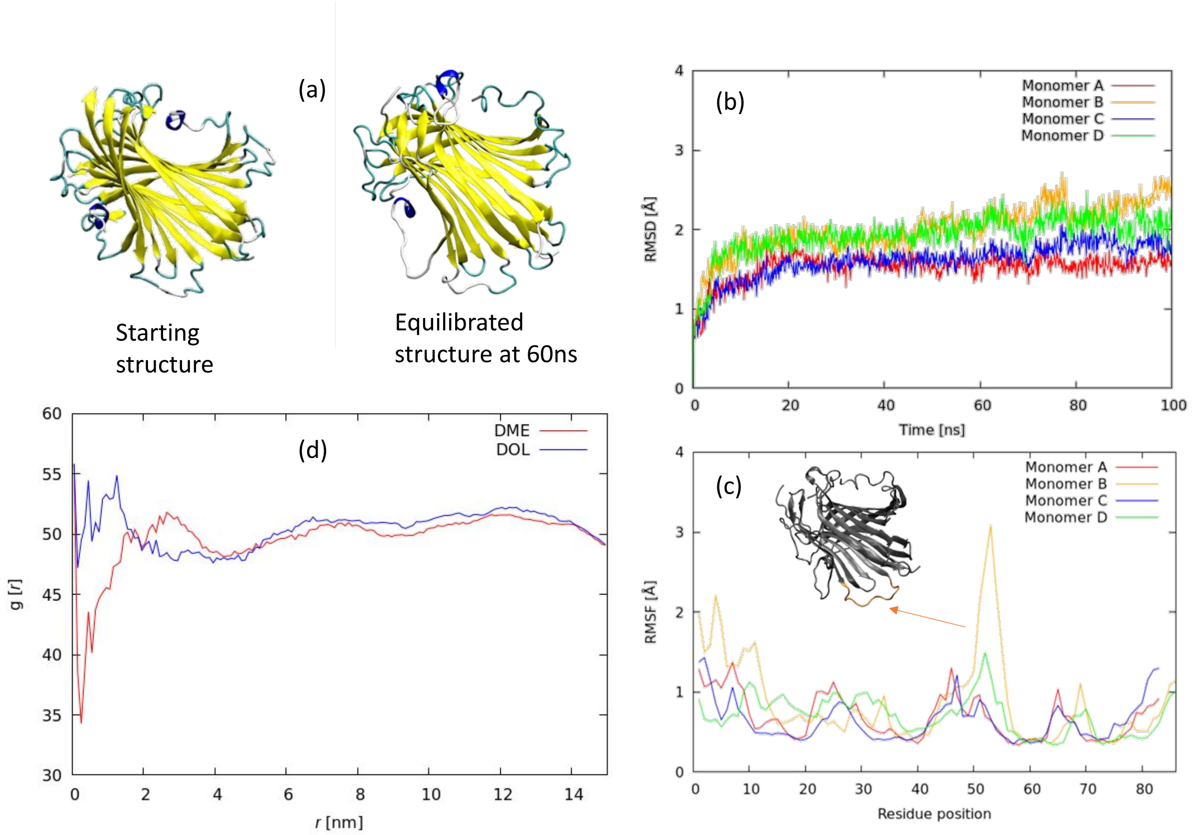

Figure SI-5. MD simulation results for System I (0.2 % w/v fibroin in DOL:DME 50:50 v/v). (a) Fibroin structure; (b) RMSD plot with respect to the starting structure; (c) RMSF plot with respect to the starting structure; (d) Center-of-mass RDF between fibroin structure and solvent molecules DME and DOL.

Figure SI-5a illustrates that the fibroin structure is more spaced after 60 ns immersion in DOL: DME compared to the initial structure, with the RMSD plot (**Figure SI-5b**) with respect to the protein starting structure showing that the average structure is reached between 20 and 70 ns, with an RMSD trend around 1.5 Å, and the RMSF plot (**Figure SI-5c**) highlighting the fluctuation around residue 50 of monomer B that is represented by a loop. **Figure SI-5d** depicts the radial distribution function (RDF), g(r), of solvents DME and DOL from the center of the fibroin structure: It can be seen that both solvents have impregnated the fibroin structure, with the DME concentrating at the fibroin center and then surrounding the structure with an outer solvation shell, forming a solvated fibroin structure of an about 8 nm diameter, whereas the main DOL impregnation and solvation shell sphere is of about 4 nm diameter. Hence, the fibroin structure is impregnated by both solvents and has a dual solvation shell comprising an inner DOL shell and an outer DME shell. Moving to the MD simulations of System II (0.2 % w/v fibroin, 1 M LiTFSI in DOL:DME 50:50 v/v), **Figure SI-6** shows that the addition of 1 M LiTFSI to System I **(Figure SI-4)** has brought a small increase of the system density and volume after equilibration, as expected.

SYSTEM II: 0.2 % w/v fibroin, 1 M LiTFSI in DOL:DME 50:50 v/v

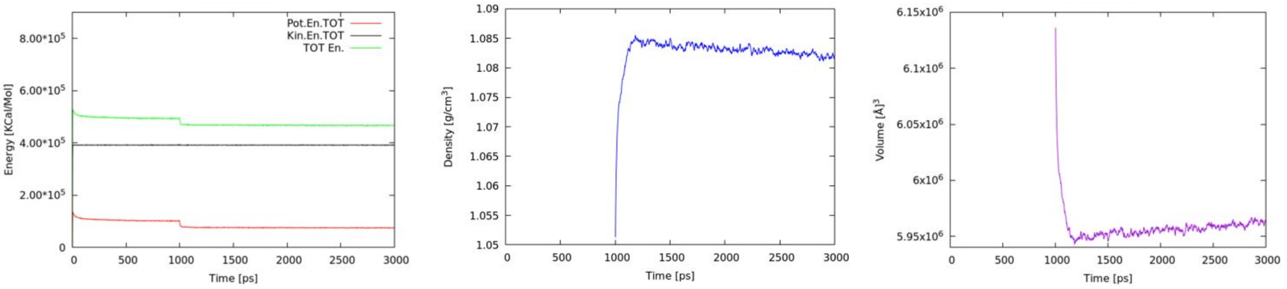

(a) (b) (c)

Figure SI-6. Plots of (a) system energies (b) system density and (c) system volume monitored during the first 1000 ps (1 ns) where the system was heated up starting from 0° to 27°C (300 K) and the equilibration step where the system was left equilibrating before starting the actual trajectory production (2000 ps).

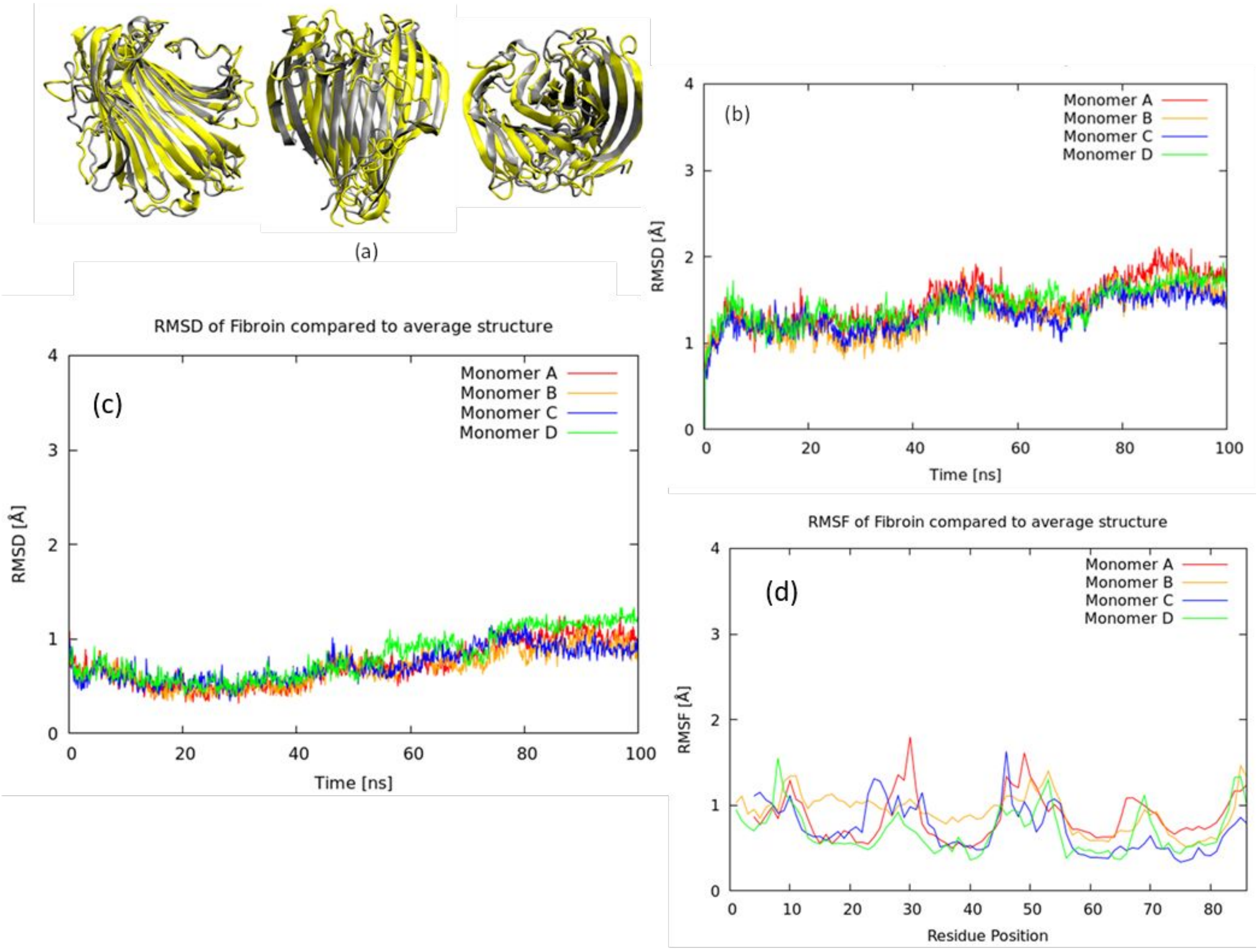

Figure SI-7. MD simulation results for System II (0.2 % w/v fibroin, 1M LiTFSI in DOL:DME 50:50 v/v). (a) Different views of the fibroin structure in System II (yellow) versus that in System I (grey); (b) RMSD plot with respect to the starting structure; (c) Plots of RMSD, and (d) Plots of RMSF of fibroin with respect to the average structure.

SYSTEM III: 0.2 % w/v fibroin, 1 M LiTFSI, 0.1 M Li_2S_2 in DOL:DME 50:50 v/v

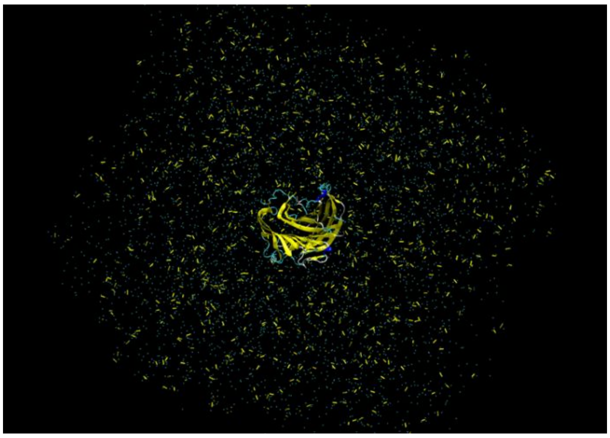

0 ns

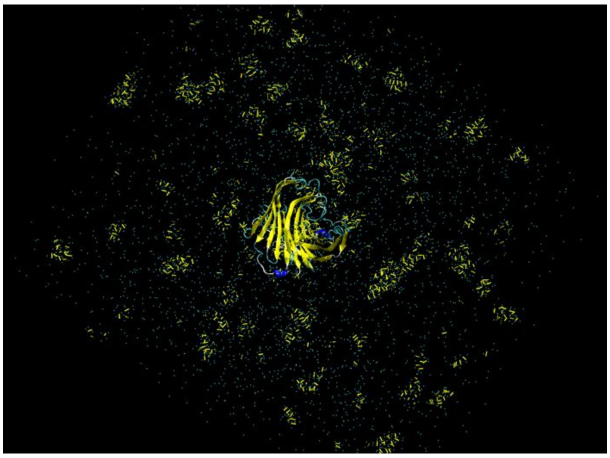

60 ns

Figure SI-8. System III simulation box depicting the silk fibroin structure and Li_2S_2 molecules at start of MD simulation (0 ns) and after 60 ns.

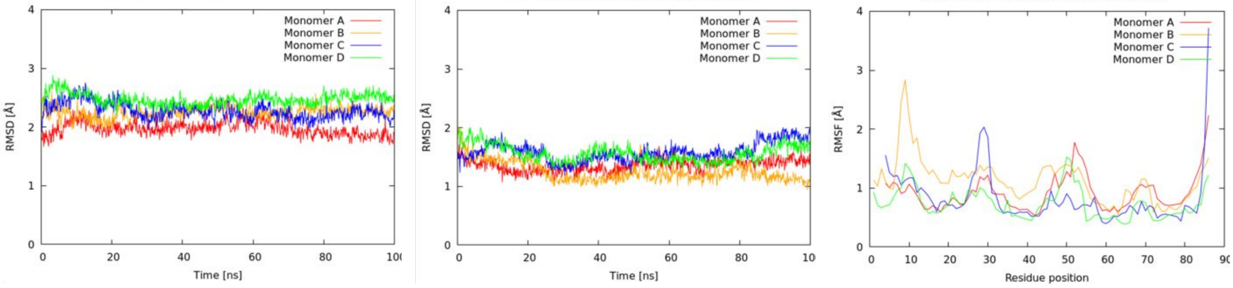

(a) (b) (c)

Figure SI-9. Plots of (a) RMSD of fibroin with respect to the starting structure and (b) RMSD of fibroin with respect to the average structure (c) RMSF of fibroin with respect to the average structure.

SYSTEM IV: 0.2 % w/v fibroin, 1 M LiTFSI, 0.25 M Li_2S_4 in DOL:DME 50:50 v/v

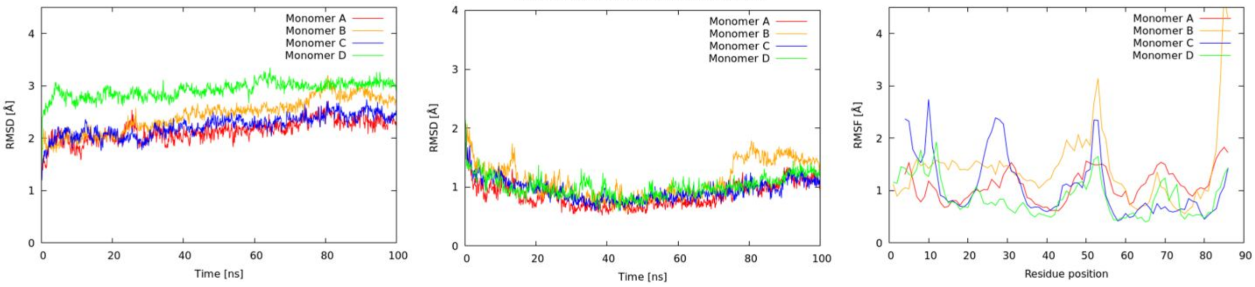

(a) (b) (c)

Figure SI-10. Plots of (a) RMSD of fibroin with respect to the starting structure and (b) RMSD of fibroin with respect to the average structure (c) RMSF of fibroin with respect to the average structure.

SYSTEM V: 0.2 % w/v fibroin, 1 M LiTFSI, 1 M Li_2S_6 in DOL:DME 50:50 v/v

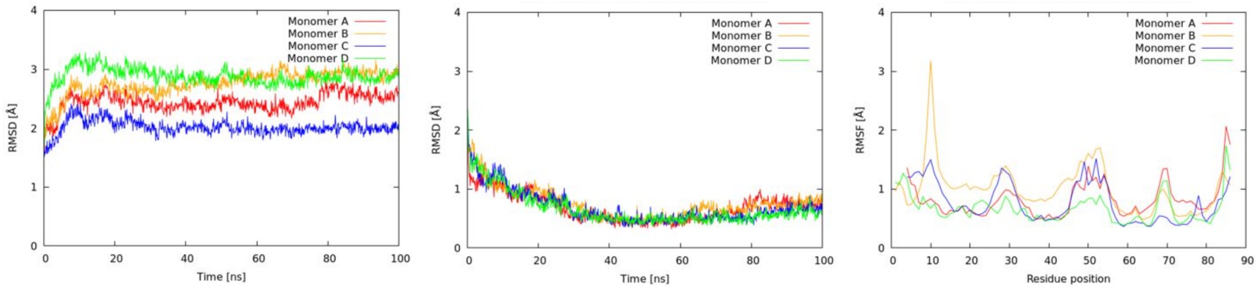

(a) (b) (c)

Figure SI-11. Plots of (a) RMSD of fibroin with respect to the starting structure and (b) RMSD of fibroin with respect to the average structure (c) RMSF of fibroin with respect to the average structure.

SYSTEM VI: 0.2 % w/v fibroin, 1 M LiTFSI, 1 M Li_2S_8 in DOL:DME 50:50 v/v

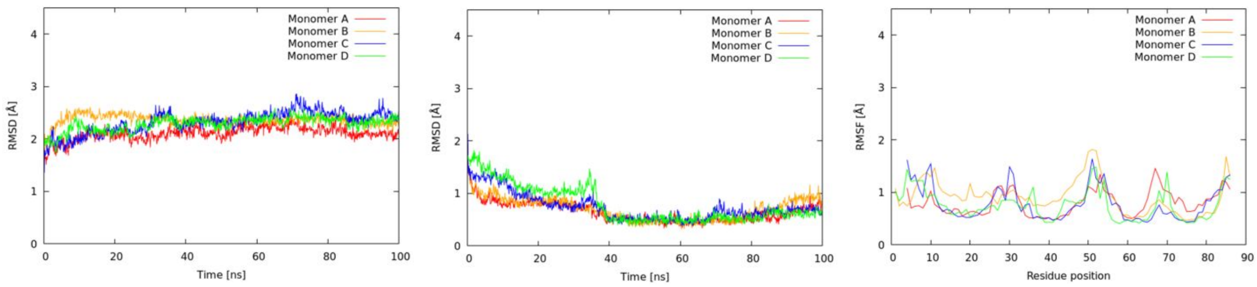

(a) (b) (c)

Figure SI-12. Plots of (a) RMSD of fibroin with respect to the starting structure and (b) RMSD of fibroin with respect to the average structure (c) RMSF of fibroin with respect to the average structure.

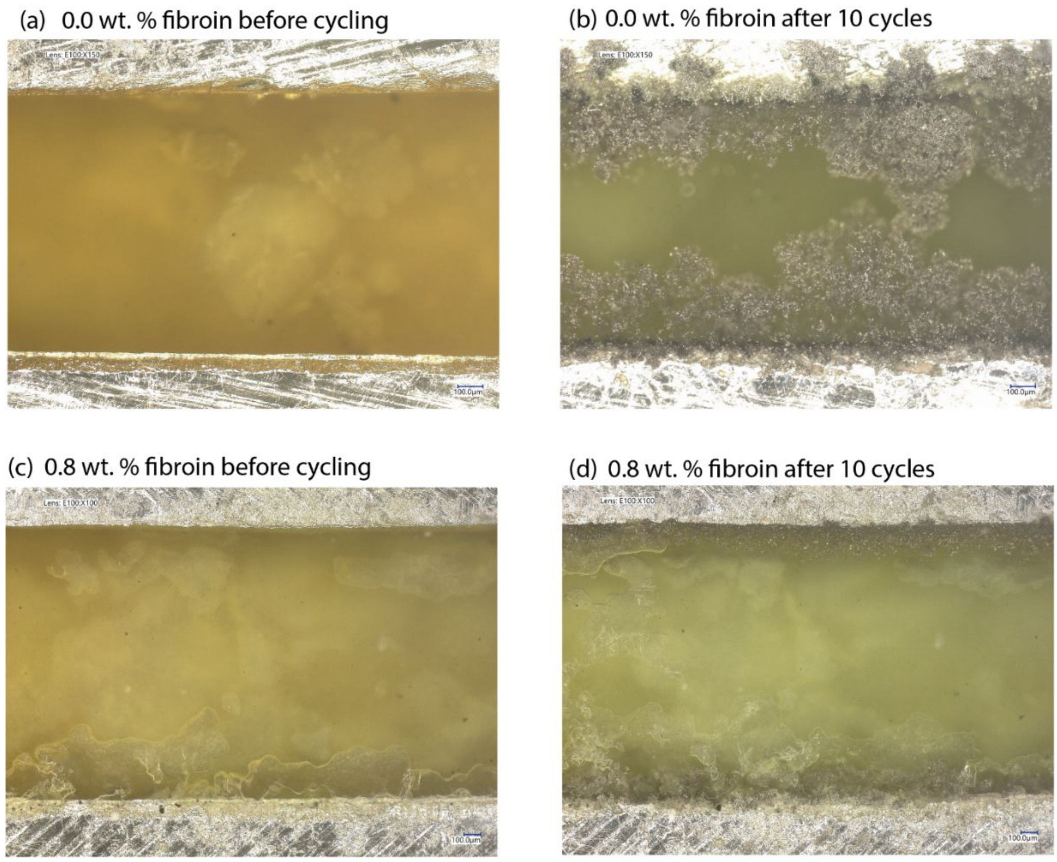

Figure SI-13. Optical images of symmetric Li||Li cells before and after 10 plating/stripping cycles carried out at current density of 3 mA/cm^2. (a) and (b) show cell with 0.0 wt. fibroin before cycling and after plating/stripping cycles, respectively. (c) and (d) shows images of cell with 0.8 wt. % fibroin before and after plating/stripping cycles, respectively. These measurements were made on El-Cell

optical cell using a Keyence VHX-7000 optical microscope. Lithium polysulfide (Li_2S_X) in a concentration of 50 mM was prepared in Li-S cell electrolyte to simulate the condition of typical Li-S cell. Further, for preparing fibroin electrolyte solution, 0.8 wt. % fibroin was added to the Li_2S_X-electrolyte through sonication.

Table SI-2. The N_{I+C} (impregnation and coordination number) of the different species with respect to the silk fibroin structure for the different Fibroin systems studied via MD simulations; all contain 0.2% w/v fibroin.

System	DME N_{I+C}	DOL N_{I+C}	Li^+ N_{I+C}	$TFSI^-$ N_{I+C}	S_2^{2-} N_{I+C}	S_4^{2-} N_{I+C}	S_6^{2-} N_{I+C}	S_8^{2-} N_{I+C}
**I**	595	881						
**II**	1346	1367	617	615				
**III**	1529	1559	641	2019	1059			
**IV**	1629	1640	980	2189		1979		
**V**	1717	2106	1021	2202			2142	
**VI**	2023	2212	1303	2225				2913

Table SI-3. The N_C (coordination number of the solvation shell) of the different species with respect to the silk fibroin structure for the different Fibroin systems studied via MD simulations; all contain 0.2% w/v fibroin.

System	DME N_C	DOL N_C	Li^+ N_C	$TFSI^-$ N_C	S_2^{2-} N_C	S_4^{2-} N_C	S_6^{2-} N_C	S_8^{2-} N_C
**I**	548	835						
**II**	1226	1300	549	545				
**III**	1410	1505	616	1902	979			
**IV**	1523	1548	942	1935		1979		
**V**	1622	2022	954	2003			2024	
**VI**	1842	2100	1217	2058				2740

The numbers in Tables SI-2 and SI-3 were calculated from the curves of g(r) versus radius r from fibroin center as a function of the system density ρ_{system} (sum of all molecules and ions/nm^3), according to the relation:

$$N_{I+C} \; or \; N_C = \rho_{system} \int_{0 \; or \; r1}^{r2} g(r) \, 4\pi r^2 \, dr \qquad \text{(S1)}$$

The N_{I+C} and N_C numbers for DOL and DME molecules with respect to the fibroin structure for Systems II-VI in Tables SI-2 and SI-3 include independent DOL and DME molecules as in System I, and some molecules solvating Li^+, $TFSI^-$ and sulfide ions, hence these N_{I+C} and N_C numbers for DOL and DME in Systems II-VI are greater than those for System I. It must be noted that Systems III-VI in Tables SI-2 and SI-3 also contain $TFSI^-$ ions from the LiTFSI salt and Li^+ ions from LiTFSI and the lithium sulfides.
